# The *Ism* between Endothelial Cilia and Endothelial Nanotubules Is an Evolving Concept in the Genesis of the BBB

**DOI:** 10.3390/ijms23052457

**Published:** 2022-02-23

**Authors:** Shireen Mentor, David Fisher

**Affiliations:** 1 Neurobiology Research Group, Department of Medical Biosciences, University of the Western Cape, Bellville, Cape Town 7535, South Africa; 2746944@myuwc.ac.za; 2 School of Health Professions, University of Missouri, Columbia, MO 65211, USA

**Keywords:** brain endothelium, tethering nanotubules, cytoplasmic projections, cilium, BBB

## Abstract

The blood–brain barrier (BBB) is fundamental in maintaining central nervous system (CNS) homeostasis by regulating the chemical environment of the underlying brain parenchyma. Brain endothelial cells (BECs) constitute the anatomical and functional basis of the BBB. Communication between adjacent BECs is critical for establishing BBB integrity, and knowledge of its nanoscopic landscape will contribute to our understanding of how juxtaposed zones of tight-junction protein interactions between BECs are aligned. The review discusses and critiques types of nanostructures contributing to the process of BBB genesis. We further critically evaluate earlier findings in light of novel high-resolution electron microscopy descriptions of nanoscopic tubules. One such phenotypic structure is BEC cytoplasmic projections, which, early in the literature, is postulated as brain capillary endothelial cilia, and is evaluated and compared to the recently discovered nanotubules (NTs) formed in the paracellular spaces between BECs during barrier-genesis. The review attempts to elucidate a myriad of unique topographical ultrastructures that have been reported to be associated with the development of the BBB, viz., structures ranging from cilia to BEC tunneling nanotubules (TUNTs) and BEC tethering nanotubules (TENTs).

## 1. Introduction

The capillaries of the brain are particularly special, as they are not simply conduits for blood, but are primarily responsible for ensuring that the neurons function in a strictly regulated homeostatic interstitium. The ability to monitor and study the orientation and alignment of brain endothelial cells (BECs) during barrier establishment is limited due to the lack of qualitative, three-dimensional, nanoscopic data. These limitations have engendered the theoretical premise that the barrier-genesis of brain capillary endothelial cells (ECs) is mainly determined by paracellular interaction demarcated by the presence of intercellular tight junctions (TJs): occludin, claudin-5, junctional adhesion molecules, desmosomes and gap junctions, all of which make up the junctional complex [1] and are known to be directly linked to the BEC actin cytoskeleton via the zonula occludens-1 plaque protein [1,2,3].

The blood–brain barrier (BBB), *in vivo*, is formed by cross-talk between the cells of the neurovascular unit (NVU) (i.e., pericytes, astrocytes and BECs). The pericytes support the angiogenic features of brain capillaries and specifically have the ability to regulate brain capillary blood flow, while astrocytes regulate BEC permeability by modulating TJ expression [1,4,5]. These supporting and modulatory cells of the NVU facilitate the BEC’s regulatory functions, which are expressed via the BEC endothelium, which regulates substance flux across the BBB.

BBB integrity is largely attributed to intercellular TJ protein interaction between adjacent BECs [6,7]. TJ protein complexes (i.e., claudins 1, 3, 5 and 12, occludin and zonula occludens -1, -2, -3 (ZO-1, ZO-2, ZO-3)) serve as intercellular paracellular gatekeepers between adjacent BECs [8]. The cerebrovasculature is deemed critical for maintaining precisely regulated CNS homeostasis, by restricting the movement of substances, ions, pathogens and inflammatory cytokines from traversing the BBB [6,9,10]. The barrier role of TJ proteins is to form an intercellular protein junction complex, which occludes the paracellular shunts. These protein–protein junctions interact by a process of dimerization [11]. Three critical transmembrane proteins are occludin (65 kDa), claudin-5 (23 kDa) and junctional adhesion molecules (40 kDa), which are linked to cytoplasmic-associated proteins ZO-1, ZO-2 and ZO-3, which interact with the BEC actin cytoskeleton and form a cytoplasmic bridge connecting TJ proteins to the cytoskeleton. Claudin-5 is the major TJ protein contributing to barrier integrity and binds homotypically to the same type of claudin (-5) on the lateral membrane of an adjacent BEC [12]. However, three cellular events are requisite for dimerization to occur: (i) BEC orientation; (ii) BEC apico-lateral nanotubule (NT) expression and (iii) BEC alignment.

Identifying structural mediators of cerebromicrovascular assembly is essential to fully comprehend the morphological landscape of barrier-genesis in the brain’s capillaries. As with all endothelia, BECs are orientated with reference to their basement membrane and are morphologically categorized into apical and basolateral domains. The emergence of a well-regulated brain capillary involves the intricacies of topographical, morphological cellular structures (e.g., nanotubules) across the BEC’s paracellular spaces, which requires the alignment of the cytoskeleton and morphology of adjacent BECs.

These structures are crucial to the developmental framework that organizes BECs to congregate and engage each other through cross-bridge topographical nanotubular networks, which engenders cellular alignment and establishes zones of TJ interaction. This review aims to illuminate novel apico-lateral NT structures as pivotal role players in occluding BEC paracellular spaces and contrast these structures with the reported “cilia” postulated to form on BECs.

### 1.1. Historical Context

The literature is scarce on the subject of BEC interaction at the nanoscopic level. However, this is not unexpected as increases in microscopy resolution are commensurate with the recent technological improvements in scanning electron microscopy. Furthermore, the use of high-resolution transmission electron microscopy (HR-TEM) has established that electron-dense intercellular regions exist in the apico-lateral regions between BECs, which have been confirmed as indicative of the zones of TJs [13,14]. However, these two-dimensional interpretations of BEC paracellular spaces under-report the complexity of the paracellular interaction. In reality, the interaction within the paracellular space is highly complex and the literature describing its dynamics remains rudimentary. During the 1960s–1970s, the successful isolation and characterization of endothelial cells (ECs) in culture was developed for routine experimentation [15,16]. In 1967, the utilization of transmission electron microscopy (TEM) by Reese and Karnovsky (1967) [17] allowed for the localization of a BEC barrier, after visualizing the inability of electron-dense tracers to traverse the paracellular spaces between adjacent BECs [17]. In the 1980s, research showed a vested interest in the characterization of ECs *in vivo*, performing differential analyses within vascular beds with respect to protein expression [18]. Based on these studies, it was established that the intact endothelium displayed both ultrastructural and molecular diversity. Although it is well established that TJ localization takes place within the apico-lateral domain of the BECs, within its paracellular spaces, how the BECs engage to form the primary barrier of the BBB to regulate transendothelial solute/ion influx from the blood into the brain parenchyma is critical for understanding CNS barrier-genesis. Compromised BEC engagement results in disruption and increased permeability of the BBB and exacerbates neurodegenerative disease progression [19] and is, therefore, a good measuring index to appraise its integrity [12]. During the 1960s–1970s, the morphology of the primary cilium was first described in fibroblastic cells [15,16]; however, it was only by the late 1990s and early 2000s that protruding cytoplasmic projections in BEC membrane surfaces were identified under both low-magnification and poor resolution (compared to current standards) and, thereafter, [2,20,21] researchers postulated these structures as endothelial cilia [22].

### 1.2. The Physiological Origin of the Endothelial Barrier-Genesis

The EC originates from the mesodermal germ layer during early embryonic development and is essential for capillary formation [23]. During the process of vasculogenesis, angioblasts originate in the lateral plate mesoderm, in the embryonic midline [24,25]. TJ protein genes are expressed in the embryonic stages of angiogenesis [26]. In mice, the BBB is formed on embryonic day 15.5 [27], and in humans, angiogenesis occurs at fetal week 8, with the BBB forming at 4 months [28,29]. Despite the presence of BEC TJ adhesion contact zones, which have been endorsed in the literature by freeze-fracture studies [30], the juxtaposed zones of TJs have to be aligned to be functional. Misalignment results in the inability of juxtaposed TJs to interact with each other to effectively seal the paracellular pathway [31]. The BEC establishes its polarity by way of basement membrane engagement, which allows for the juxtapositioning of the apico-lateral region of the paracellular spaces between adjacent BECs, resulting in aligned zones of TJ interaction and subsequent occlusion of the paracellular space, creating barrier separation between the inside and the outside of the brain’s microenvironment. The apical membrane, which is of interest in this review, is positioned towards the external milieu/capillary lumen and there have been reports postulating the presence of cilia on the apical membranes of endothelial cells [2].

These early studies have postulated the role of endothelial cilia in either capillary flow dynamics or the genesis of the BBB [32]. These studies suggest that cilia exist on BECs and that this is functionally involved in angiogenesis and the regulation of blood flow. A landmark study by Mentor and Fisher (2021) [31] evaluated the topographical landscape of BECs in barrier formation, elucidating which topographical structures play a role in facilitating the alignment of the paracellular spaces between adjacent BECs. However, cilia were conspicuously absent. In contrast, this is the first morphological evidence that strongly suggests that NTs play an important role in BBB-genesis. Thus, we address the schism between the high-resolution scanning electron microscopy (HR-SEM)-based evidence of endothelial NT generation observed during EC monolayer development, which provided insight into the development of the brain capillary, and compare this evidence to the immunofluorescence and molecular evidence underpinning the postulate for BEC “cilia”.

## 2. Nomenclature Clarifying Morphological Structures

The nomenclature of tubular structures has produced lots of confusion in the use of terminology for structures that extend from the plasma membrane. We, hereby, attempt to address this by describing and defining clearly the structures filopodia, cilia and the different types of NTs.

### 2.1. Filopodia

**Figure 1:**
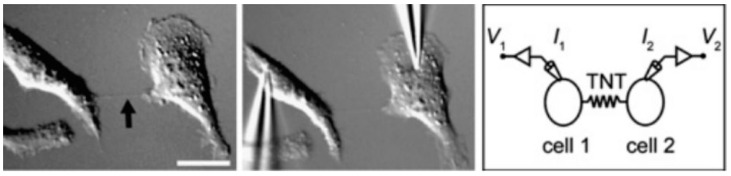
A micrograph illustrating filopodial “TNT” (tunnelling nanotubule) extensions between two normal rat kidney (NRK) epithelial cells (black arrow) (**Right**); during electrophysiological recordings (**Left**). Scale bar = 20 μm. *V*_1_ and *V*_2_ denote voltage applied to cell 1 and cell 2, and *I*_1_ and *I*_2_ denote the current injected into cell 1 and cell 2. These experiments indicated that filopodia electrically connect cells [37].

Historically, cytoplasmic protrusions emerging from cells were denoted as filopodial extensions between adjacent cells. The term filopodia has its roots in the term pseudopodia, implying “false-feet”. The filopodia, when broken down, implies a family of a specific type of foot-like structure, viz., an extension from a parental body (i.e., the cell) [33]. The filopodia are thought to be involved in the migration of the BECs crucial for the repair of the capillaries (viz. as seen in the *in vitro* scratch assays, or following an *in vivo* cerebrovascular accident). To date, filopodial structures have sometimes been incorrectly associated with tunnelling nanotubule (TUNT) formation [34,35,36]. In terms of dimensions, the size of filopodia is in the micrometer range (80 µm in length; see Table 1) and, therefore, by definition, they should not be categorized together with nano-sized morphological structures. It is, therefore, incorrect to refer to these structures as “nano”-tubules, as seen in Figure 1.

An electrical coupling study, reported in NRK cells, illustrated the ability to apply an electrical voltage to one cell and monitor its ability to conduct a current from cell to cell, across filopodia (TNTs), using patch-clamping techniques, suggesting that cells are able to communicate via these filopodia using electrical signaling (Figure 1). Furthermore, the filopodia are f-actin-based intercellular conduits that play a functional role in direct intercellular communication, spanning lengths of 10–80 μm. These filopodial-like f-actin-rich protrusions form intercellular cross-bridge networks. This was indeed the first tubular structure proposed that was reported to be involved in cell–cell interaction [34]. A study by Dieriks et al. (2017) [38] reported that filopodia possess a “cargoing” function between *in vitro* neuroblastoma cells implicated in Parkinson’s disease (i.e., SH-SY5Y, human cell line). Furthermore, work describing the role of filopodial-like structures in brain tumors in mice showed the functional importance of tubular connections *in vivo* [38,39]. BEC filopodial structures are typically seen between cells grown on glass or in a Petri dish and, in terms of dimensions, are fundamentally in the micrometer range (see Table 1) and function primarily in terms of intercellular communication or migration.

**Table 1:** Comparison between filopodial and BEC NTs.

	Filopodia	BEC NT
Size	µm	nm
Diameter	200–400 nm [40,41]	50–100 nm [31]
Length	80 μm [42]	<1000 nm [31]
Location	Basolateral domain of cultured mouse melanoma cells [43]	Apico-lateral domain of cultured mouse BECs [31]

### 2.2. Cilia

Between the late 1990s and early 2000s, protruding cytoplasmic projections in BEC membrane surfaces were identified and, thereafter, [2,20,21] postulated these structures as endothelial cilia [22]. These reports stated that these endothelial cilia-like structures may be critical for vascular remodeling upon identifying cilia-like structures during vascular development (i.e., vasculogenesis and angiogenesis). However, how do we identify membrane protrusions as cilia?

**Figure 2:**
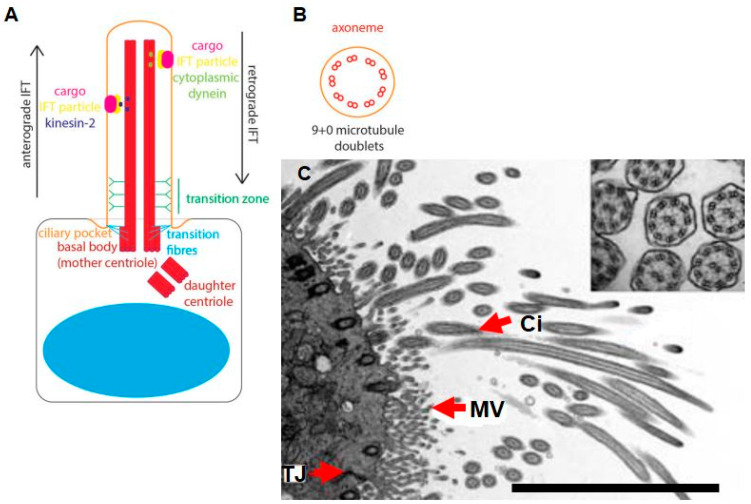
A schematic illustration of cytoplasmic protruding cilia, originating from the cell centrosome. (**A**) A typical primary cilium projecting from the cell surface, comprising cargo, viz., intraflagellar transport particles: kinesin-2 and cytoplasmic dyein; (**B**) a cross-section of a non-motile cilium, which assumes a 9 + 0 formation of microtubule doublets [16]; (**C**) a TEM image of tracheal epithelium and a cross-section of tracheal cilium displaying a 9 + 2 conformation. Ci denotes cilia, MV denotes microvilli and TJ denotes epithelial tight junctions. Scale bar = 8 μm [50].

By definition, the archetypical cilium is an apico-lateral-based structure, with a highly structured set of microtubules. These internal cytoskeletal microtubules are easily identified by TEM (see Figure 2). The typical conformation of its axoneme is constituted by nine pairs of post-translational, acetylated peripheral microtubules, which are arranged according to its motility status. Non-motile, primary cilia are present in mammalian cells (i.e., fibroblasts, epithelial and muscle cells) [44], which have a 9 + 0 arrangement of microtubules within their axoneme; conversely, the motile cilia contain a 9 + 2 microtubule arrangement [2,21,45,46,47]. Generically, the cilium is rooted at its base by a basal body, which is derived from the centriole of the centrosome [48], the latter structure being essential for nucleating the mitotic spindle during cell division. During mitosis, the cilium is resorbed to release the centrioles, and cilio-genesis commences after the completion of cytokinesis [16,49] (Figure 2).

The authors of Figure 2A–C give a strong rationale for defining cilia, namely that they have clearly seen this conformation via HR-TEM and thus have ascribed this function and structure to the cilium, based on micrographical data. Many differentiated mammalian cells have been reported to produce primary cilial extensions, which possess chemosensory and mechanosensory functions to respond to external stimuli, and thus are classified as organelles that function in integrative signaling from extracellular signals, promoting physiological functioning within cells [2,45,51,52]; however, to date, the exact nature of ciliary mechanosensory functions in BECs remains moot as no empirical data have been supplied to support this premise. The proverbial “elephant in the room” is that no one has reported an HR-TEM micrograph of the cytoskeletal structure of a BEC cilium. Given the extensive occurrence of immunofluorescence “evidence” reported for BEC cilia, it should be routine to use HR-TEM to identify BEC cilia.

### 2.3. Nanotubules

In contrast, nanotubules (NTs) are involved in cell–cell interaction across the paracellular spaces between adjacent BECs and, in terms of dimensions, are less than 1000 nM, and they are involved in the mechanical stabilization and alignment of the paracellular space, as well as intercellular communication. Before the study by Mentor and Fisher (2021) [31], existing reports of cytoplasmic projections failed to resolve the role of cytoplasmic-based NT projections between BECs during BBB formation. This study described the BEC NT as an expressed topographical structure on the apical surfaces of BECs. Furthermore, two novel NTs were described: nanovesicle (NV)-derived tunnelling NTs (TUNTs) and rope-like tethering NTs (TENTs), which extend across the paracellular spaces between adjacent BECs during monolayer development. The TENT plays a crucial role in aligning adjacent BECs to facilitate the interaction of TJ zones between juxtaposed lateral BEC membranes and promotes cell–cell hemifusion/TJ interaction. Moreover, TENTs play a key role in the formation of the typical overlapping apical membrane regions of BECs, which shield TJ loci and reinforce paracellular occlusion, subsequently contributing to BEC monolayer integrity [31].

Moreover, BECs possess NVs, which are extruded onto the surfaces of BECs growing in close proximity. Some of these NVs are extruded from the cell membrane and possess a specific surface topography that is distinctly different from the plasma membrane. These NVs display a propensity to fuse together, forming a tube (tunnelling-nanotubule: TUNT) between BECs, which connects the lateral membranes of two adjacent cells. The significance of these NVs is that they are hollow and devoid of cytoskeletal structures. It is, therefore, inferable that the NVs possess the same molecular contents, which are involved in intercellular signaling processes, triggering the same morphological/molecular signals, which bring about BEC alignment during brain capillary endothelial development [31]. The NVs are thus secreted to form tubes, which connect two adjacent cells, with their ends incorporated into the BEC membrane, providing identical signaling between cells.

### 2.4. Postulational Brain Endothelial Cell Primary Cilium

Cilia are reported to be associated with quiescent cells, while cells that are involved in the cell cycle are non-ciliated (this is because the basal body of cilia forms the centrosomal bodies during cell division, and only after cells enter their quiescent phase do these centriole-derived basal bodies become available for ciliogenesis). Cilia are described as hair-like and/or flagellar structures that form on the cell surfaces of eukaryotes through the process of ciliogenesis [47,53]. Postulated cilia of the vascular endothelium are reported to extend into the lumen of the blood vessel and respond to sensory stimuli (i.e., extracellular stimuli) [29,45]. These “primary cilia” are described to be functionally involved in vascular barriers by exhibiting a sensory function that allows for the transmission of extracellular signals into the vascular endothelial cell, contributing to blood vessel function, through sensing blood flow and cell migration [2,22,32,45]. The index by which to conclude that an extracellular organelle extending from the plasma membrane of a BEC is in fact cilia requires that the following criteria be taken into consideration: is it an extracellular organelle adjoined with the plasma membrane, and are these extensions structured with a 9 + 2 or 9 + 0 microtubule cytoarchitecture; are these structures associated/anchored with a basal body and are they motile and/or sensory? [54,55]. Transmission electron micrographs support the presence of (9 + 0; 9 + 2) microtubule doublets in cross-sections of tracheal, intestinal and bronchiole epithelia [56]; however, evidence of this microtubule conformation in BECs has yet to be observed or reported in the literature.

**Figure 3:**
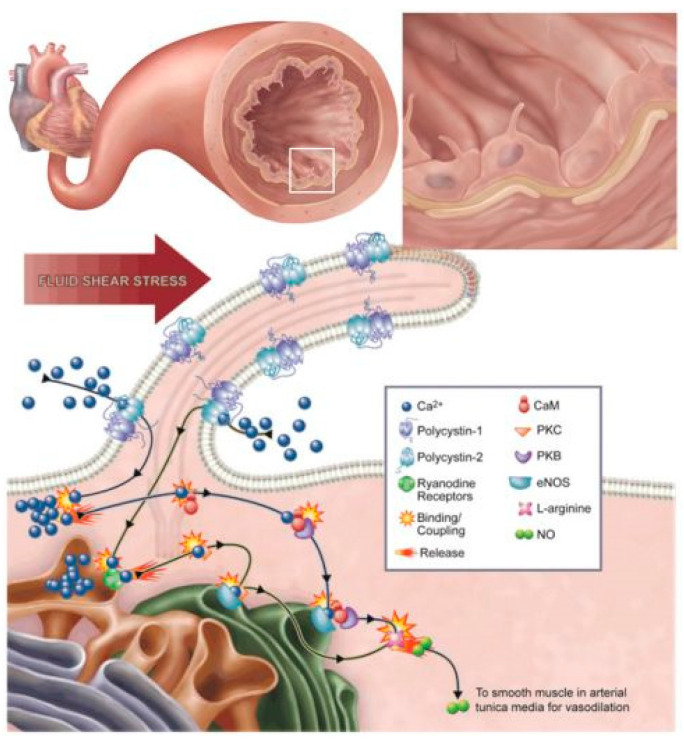
An illustration postulates endothelial primary cilia as regulators of blood pressure through nitric oxide production, which is transferred to the smooth muscle in arterial tunica media [51].

Mohieldin et al. (2016) [51] reviewed primary cilia, postulated in blood vessels of mouse arteries and blood vessels of human patients (Figure 3).

In this review, the authors [57,58] postulate a mechanosensory function for primary endothelial cilia that is due to polycytin proteins (i.e., polycytin-1 and polycytin-2), which are reported to respond to changes in blood pressure or shear stress within the blood vessels, triggered by changes to the influx of calcium. The study, however, fails to concretize these postulates with empirical data (i.e., graphical and/or micrographical findings) and, thus, the role for BEC cilia has not been supported with concrete data. Endothelial cilia in cardiac arteries thus remain a postulate.

Postulated EC cilia have also been suggested to play a role in extracellular fluid mechanics in an intracellular signaling cascade, which activates endothelial nitric oxide synthase (eNOS) and results in vasodilation [51]. Furthermore, with respect to blood pressure and blood flow dynamics, it is well established that sphincters within the walls of arterioles and pre-capillary arterioles regulate the flow of blood through capillaries using neural and local mechanisms of vasodilation and vasoconstriction [59,60], which is driven by the eNOS system and has never been linked to cilia’s mechanisms of action. One critical function of the BEC is to regulate the flux of substances across the BBB; thus, it is unlikely that the primary cilium is at the nexus between fluid dynamics and vessel dilation of the brain’ capillary ECs as the capillary lacks contractile elements and thus is not involved in modulating blood vessel diameter. The function of capillary diameter regulation is not the role of the endothelial cell, but rather the role of the pericytes of the BBB. This is well established in the literature [61].

**Figure 4:**
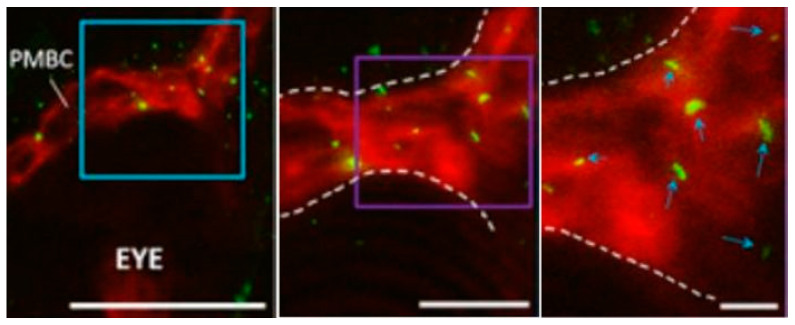
Determination of “cilia” distribution on EC membranes of primordial cerebral vessels. Confocal micrographs of GFP-labeled primordial midbrain channel in transgenic line *Tg (kdrl:mCherry-CAAX)^y171^* and *(bactin::Arll3b:GFP)* to help label EC membranes. Scale bar = 40 μm [22].

Eisa-Beygi et al. (2018) [22] showed the emergence of cilia in early cranial vessels assembling during angiogenesis in hindbrain capillaries in a study utilizing zebrafish. “Cilia” distribution was seen in ECs upon intercrossing several tissue-specific transgenic reporter lines (i.e., *Tg(kdrl:mCherry-CAAX)^y171^* (Figure 4) [62], which enabled the labeling of EC membranes to demonstrate the distribution of EC cilia [40]. The aim of this study was to measure flow velocity and shear stress. After 24 h, green fluorescent protein (GFP)-probed cilia were observed throughout the blood vessel, within the primordial midbrain channel, predominantly accumulating at the boundaries of intravascular spaces (Figure 4).

**Figure 5:**
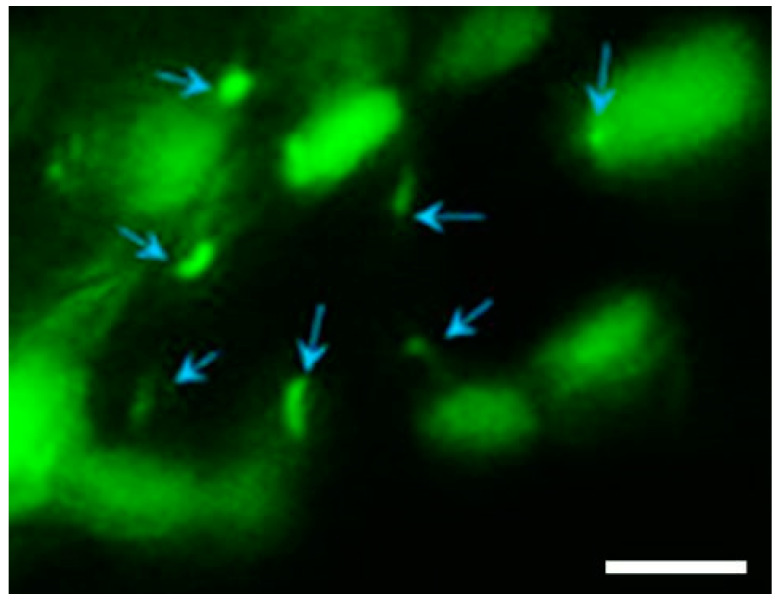
Photomicrograph of primary cilia, denoted by blue arrows. An illustration of GFP-labeled cilial extensions within primordial midbrain channels. Scale bar = 5 μm [22].

Furthermore, low-resolution imaging was performed, utilizing confocal microscopy to visualize the characterized distribution of cilia in BECs. “Cilia” were found to be distributed around the edges of the cell, projecting into the intravascular spaces (Figure 5 below).

GFP refers to the gene that produces green fluorescent protein. The authors suggest that structures labeled with GFP are cilia (Figure 5) [22], which play a functional role in the early stages of cerebral–vascular morphogenesis; however, biologists normally use GFP as a generic protein marker. GFP can attach to and mark proteins with fluorescence, enabling scientists to see the presence of the particular protein in an organic structure. This type of fluorescent marking cannot explicitly identify cilia per se. This is evident as much more than just the “cilia” has been tagged with GFP fluorescence (Figure 5). Control of EC behavior and morphology is critical during vascular formation and remodeling; however, at such a low resolution, it is implausible to clearly identify a distinctive cytoskeletal profile of cilia. Furthermore, at this resolution, it is also unclear whether one can show the 9 + 2 cytoskeletal structure of the cilium, and supporting evidence from TEM microscopy has never produced proof to suggest the presence of endothelial cilia. What is clear is that GFP fluorescence extends from the soma of the BEC into these projections. This indicates that the protein-based cytoskeletal structure of the BEC projects into the tubular projection. This cytoskeletal immunofluorescence (IF) has also been described for TENTs in BECs, but, in the case of TENTs, clear HR-SEM micrographs support the IF-based evidence for TENTs.

GFP staining does not emphatically make a cytoplasmic projection a cilium—it simply implies that it is a tubulin-based structure and not “cilia” *per se*. Furthermore, nowhere do we see presented evidence of a 9 + 0/9 + 2 cross-sectional conformation.

**Figure 6:**
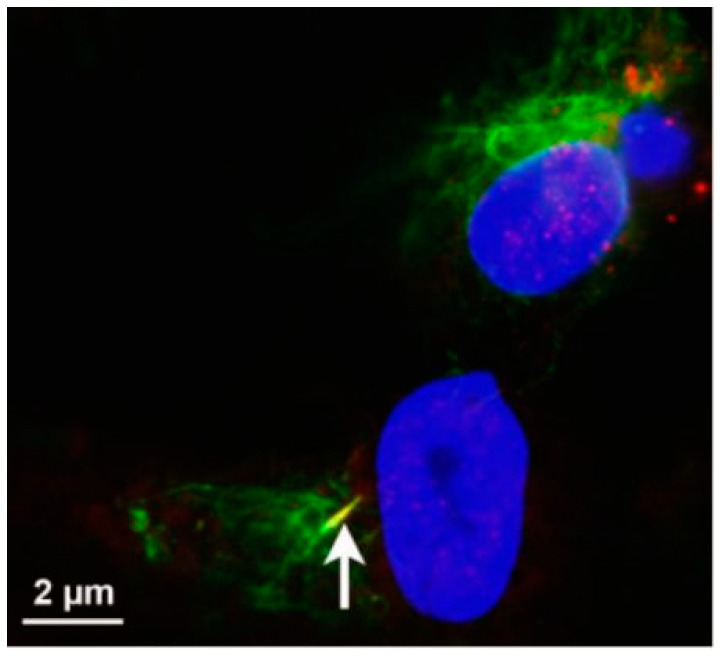
Immunofluorescence (IF) illustration of EC “cilium-like structures”. The white arrow exhibits a gamma-tubulin AC3-positive tubular structure, which is postulated to be a cilium [52].

Moreover, a study by Antal et al. (2017) [52] reported on mammalian cells possessing a primary cilium, which is generated during growth arrest of the cell. It further presumed that many single-layered epithelia possess a primary cilium, excluding the small intestine and the colon. Moreover, adenylate cyclase type III (AC3)-positive cilia were reported to be found in cells of mesenchymal origin, namely smooth muscle cells and ECs. AC3 is an enzyme involved in the synthesis of cyclic adenosine monophosphate from adenosine triphosphate and is found on the plasma membranes of the neuronal primary cilium. It was first reported to be present on olfactory neurons and has since been found in endothelial cells *in vitro* (Figure 6), which is in addition to the hypothesis that primary cilia may play a role in the regeneration of select mesenchymal cells. However, to date, there have been little to no postulations on the potential role of AC3-positive cilia in BECs. AC3-based identification of cilia needs to be corroborated by additional evidence, viz., HR-TEM, as, on its own, it does not prove the presence of cilia.

The “cilium” in Figure 6 was identified using immunocytochemistry, by studying the molecular dynamics of the cytoskeletal protein tubulin. The authors propose that an EC is able to display a singular acetylated tubulin-positive cilium. It is presumed that cilia are engendered from tubulin, which extends between two adjacent ECs [52].

**Figure 7:**
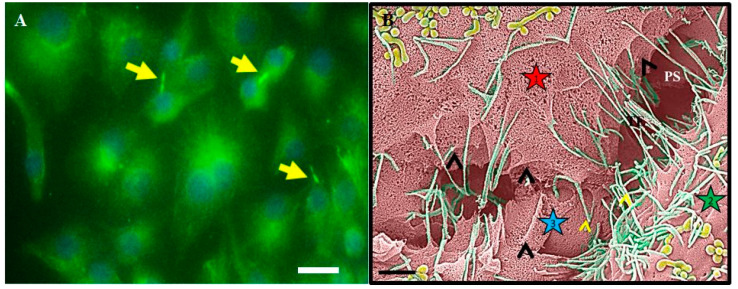
The cytoarchitectural organization of primordial cytoplasmic projections. (**A**) The formation of TENT extensions by cytoplasmic extension of α-tubulin in BEC bEnd5 cells, from the BEC leading membranous edges. The yellow arrows indicate TENT extensions, scale bar 20 μm [66]. (**B**) TENT formation on the apico-lateral surfaces of the BEC bEnd5 plasma membrane [31]. PS denotes the paracellular space; the stars represent cells 1, 2 and 3. The black arrows indicate areas of continuous membrane leading edges of BECs, governed by cytoplasmic projections, which develop into thin rope-like tethers. Scale bar 1000 nm.

Based on the cytoarchitectural dynamics of TENT structures, it is highly debatable that tubulin, which is ubiquitous within the cell’s cytoskeleton, results in a single cytoplasmic projection. Based on the molecular dynamics of tethering NTs (TENTs), tubulin extends directly into the membranous protrusions from the BEC plasma membrane surface into TENTs between adjacent BECs (see Figure 7). In Figure 7, HR-SEM findings strongly suggest that focusing on a single projection negates the vastly complex physical functionality of cytoplasmic projections, which concentrate along the BEC membrane’s leading edges, facilitating cell–cell engagement during BEC monolayer formation.

In addition, the study by Mohieldin et al. (2016) [51], suggests that the presence of primary “cilia” correlates with the onset of angiogenesis, suggesting that “cilia” are critical in the early processes of new blood vessel formation and damage to the “cilia” induces an array of vascular diseases [22,51,63]. Despite references that allude to the functionality of the EC primary cilium, namely its mechanosensing ability at the blood–tissue interface [45], the notion that BECs of cerebromicrovascular beds possess cilial-like structures within their lumen is difficult to conceive, as capillary pressure is directly proportional to blood pressure, which drives blood flow through the capillary, and thus the postulate that cilia on BECs are responsible for sensing/regulating capillary blood flow simply introduces an entirely new dynamic and/or obstruction to the functionality capillary flow dynamics that is not currently supported by empirical data. Nowhere do these postulates on ciliary function and blood flow of shear stress address the role of the pericyte in regulating local capillary blood flow.

A study by (Ma and Zhou et al., 2020) and (Kallakuri et al., 2015) [35,64] reported that “cilia” are present in the vascular ECs of zebrafish brains; however, in the absence of high-definition morphological clarity around these structures, it is understandable how its description of cilium structures could be misconstrued. These presumed cilia structures are likely being misidentified. Given these observations, it may be of interest to re-evaluate the data that have postulated the presence of EC “cilia” in light of new HR-SEM-based evidence in BEC endothelia [31].

Given the contention that these endothelial structures may be “cilia”, it is important to note that, in the literature, there is a wide array of actin-based NT structures, which are denoted as TENTs, intercellular cross-bridges, NT highways and cytoplasmic projections. The most widely reported function of the NTs is their ability to cargo proteins, DNA, RNA, organelles and viruses [65]. However, the physical functionality of NTs in BBB construction is described for the first time in the *in vitro* BBB model [31,66]. The study addresses the functional role of actin and tubulin in the cytoskeletal structure of the TENT [66].

### 2.5. TENTs

In a recent HR-SEM study by Mentor and Fisher (2021) [31], numerous cytoplasmic projecting nanostructures, denoted as BEC nanotubules (TENTs), were described (Figure 7A,B). In these studies, NTs are further divided into two membranous extensions: (i) tunneling NTs (TUNTs) and (ii) tethering NTs (TENTs) [31]. In contrast to the extrapolated evidence for postulated endothelial cilia, the authors use HR-SEM [31] to depict a highly dynamic set of apical and apico-lateral nano-projections from the BEC membrane. These high-definition photomicrographs have led to the postulate that tunneling NTs (TUNTs) and tethering NTs (TENTs) exhibit an ability to facilitate the alignment and localization of TJ proteins between adjacent BECs in culture, which is the hallmark of BBB establishment [31]. Tethering nanostructures are of interest in this review as they are observed extensions from the plasma membranes of BECs. These overlapping, tent-like structures progress to form slender ropes that play a role in the occlusion of the paracellular shunt between adjacent BECs and were, thus, denoted as TENTs. TENTs are observed as cellular protrusions, which are continuous with the apical and apico-lateral regions of BEC membranous leading edges and are, thus, extensions of the BEC phospholipid bilayer [28]. Furthermore, in a study by Mentor et al. (2022) [66], it was found that the ultrastructural TENT is governed by select cytoskeletal proteins (i.e., f-actin and α-tubulin). These findings endorse the influence of the TENT on endothelial barrier-genesis as the cytoskeleton is directly linked to the junctional and plaque TJ proteins, strongly suggesting that TENTs are critical in the alignment and interaction of BEC–cell junctions to form a well-regulated vascular barrier.

In the study by Mentor and Fisher (2021) [31], BEC TENTs were qualitatively evaluated using HR-SEM to generate a three-dimensional map to investigate its morphogenesis during BEC monolayer establishment. Furthermore, a supporting study by Mentor et al. (2022) [66] utilized depolymerizing agents (Cytochalasin D and Nocodazole) to suppress the expression of cytoskeletal proteins f-actin and tubulin during BEC monolayer development, using immortalized mouse BECs (bEnd5) as an *in vitro* BBB model. In this study, interactions between adjacent BEC membranes were suggested to be facilitated by f-actin-rich microfilaments and α-tubulin-rich microtubules and were further proven to be the intracellular backbone of BEC TENTs [66].

The discovery of the TENT creates the contention between postulated EC cilia and ubiquitous BEC NTs. In view of the discovery of the BEC TENT, we question if indeed cilia are found on the surfaces of BECs, as a separate class of cytoplasmic projections. Despite having studied thousands of BECs under high-resolution microscopy and finding no evidence for apical cilia, one cannot exclude the possibility that they exist. However, if they are integral to the physiological function of the BEC or the brain capillary, then routine observation should clearly lead to their observation and, currently, this is not the case. Secondly, transmission electron microscopy (TEM) studies have been carried out on BECs for decades, leading to clarification of the molecular occlusion of the paracellular space by TJs, yet none have identified cilia. Even though the absence of cilia identified by HR-TEM/HR-SEM does not preclude their presence, it is essential that collaborated evidence is obtained before we start to ascribe postulated functions to these low-resolution, low-magnified structural extensions from the soma of BECs.

In Figure 7, cytoplasmic extensions are pervasive within BEC paracellular spaces, forming cell–cell networks and resulting in BEC membrane juxtapositioning, which further promotes TJ localization and the establishment of a highly restrictive BEC monolayer [66]. It is noteworthy, at this stage, to compare our documented IF micrographs of TENTs with those described in Figure 5 and Figure 6 as cilia. These same NT structures seen with HR-SEM (Figure 7B) clearly do not depict “cilia”.

The cytoplasm is a polyphase material and its dynamic nature has been insinuated to be central to the cytomechanics of cell shape, migration and division [67]. Furthermore, the role of cytoskeletal elements in TENT formation is a critical aspect of cytoplasmic modifications during BEC monolayer development. Based on the empirical findings in Figure 7 [66], TENTs are α-tubulin- and f-actin-rich tethering structures.

### 2.6. The Role of I-BAR Proteins’ Nano-Tubular Formation

The role of f-actin in “membrane shaping” is key to cellular processes such as transcytosis, cell division, filopodial protrusions and NT formation [31,34,36]. The formation of membranous structures is endorsed by the role of regulators of membrane curvature, which is a family of proteins that comprises the crescent-shaped Bin/Amphiphysin/Rvs (BAR) domain [68].

**Figure 8:**
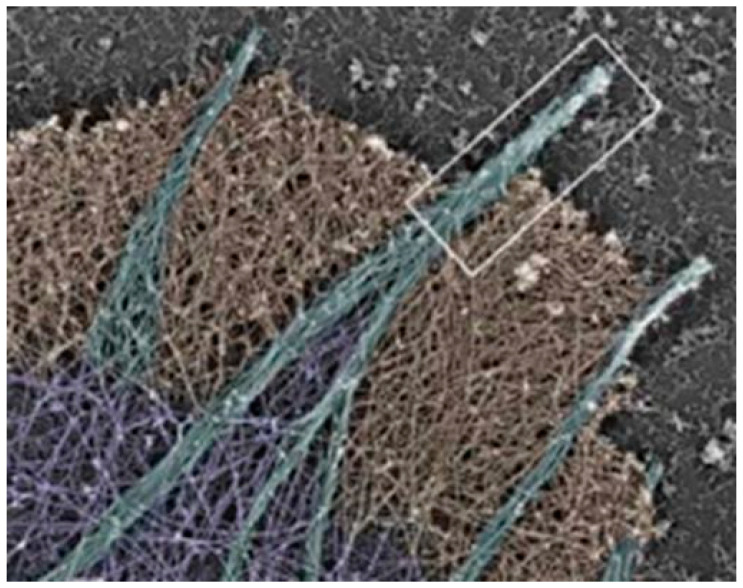
Electron micrograph of actin-based membranous protrusions from leading edges of cultured mouse melanoma cells as denoted by the white rectangle [43].

I-BAR domains cause membranes to inversely/negatively curve towards the extracellular environment. I-BAR domains contain the actin-bundling protein with BAR domain-containing adaptor protein 2 (BAIAP2) homology (i.e., referred to as ABBA protein), which is associated with membrane protrusions. Furthermore, I-BAR domains have been reported to produce tubules within a range of 40–80 nm in diameter in *Escherichia coli* BL21 (DE3) cells [69]. The cell membrane, thus, becomes curved, allowing for (i) tubular carriers (i.e., lamellipodia/filopodia) and (ii) TENTs/TUNTs to form from flat membranes (Figure 8). These postulates are supported by the polymerization of actin polymers that are closely associated with protrusions that bring about cell membrane extensions [43,70].

The filopodia are rich in actin and their protruding extensions and are governed by cell membrane deformation by the I-BAR proteins, resulting in the negative curvature of the membrane away from the cell’s cytoplasm [43].

**Figure 9:**
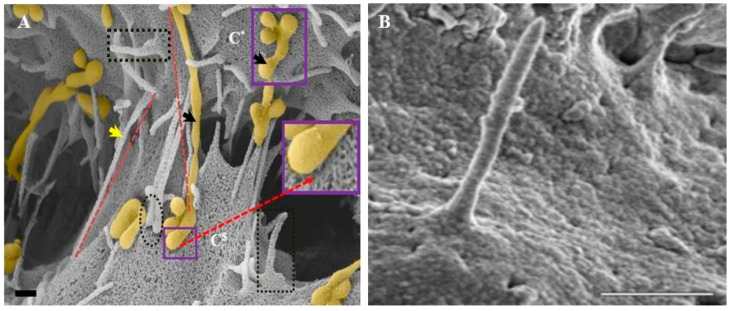
TENT development on the apical surface of BEC bEnd5 plasma membrane surfaces. (**A**) Early/primordial TENT development on the plasma membrane surface of the BEC at high magnification, represented in the black perforated boxes. C* denotes the membrane of cell one, C^$^ denotes cell two, the yellow arrow denotes a fully formed TENT structure extending across the paracellular space between two adjacent BECs and the black arrows denotes nanovesicle-induced TUNT structures. Scale bar = 200 nm [31]. (**B**) A SEM photomicrograph of proposed primary endothelial cilium at high magnification. Scale bar = 1 μm [71]. Note the similarity between the primordial TENT (perforated square) in (**A**) to the postulated cilium in (**B**).

Synthesis:

It appears that the TENT and “cilia” possess identical entrails of microtubule conformation, which begs the question, “were the postulated cilial structures actually developing primordial TENTs?” Given the low-magnification and low-resolution of these postulated cilia, was it simply a case of misinterpreting the visual and fluorescent data? The overall structure, function and localization of these two cytoplasmic variants could likely be narrowed down to cytoskeletal semantics. TENTs are inclined to accrue and develop on the apical and/or apico-lateral plasma membranes of BECs and, in their early stage of development, resemble cilial structures. However, they mature into cytoplasmic projections, which form a tethering scaffold across the paracellular space of adjacent BECs during monolayer development, which is crucial for membrane alignment, interaction and the consequential occlusion of the paracellular space. Little is known about the sequential development of cilia structures. Moreover, given the HR-SEM evidence on TENTs, it has become clear that the TENTs are abundant on BECs during monolayer development. According to fluorescent-based observations, postulated cilia should be abundant on the apical surface of BECs, but when studied using either HR-TEM or HR –SEM, this is not observed. This critical review aims to show the contrast between primordial TENTs and structures identified largely through IF or molecular studies, which are postulated as “cilia”. Both the postulated cilia and the TENTs are projections from the BEC apico-lateral membrane surface. Both have cytoskeletal structures, which include f-actin and/or tubulin. However, actual HR-SEM and HR-TEM evidence does not suggest that these structures are cilia, as they have no defined cilia microtubular cytoskeletal structure (9 + 2). Given that primordial TENTs, or newly developed TENTs, are short f-actin/tubulin extensions and that the evidence for cilia consists of mostly IF observations, it is easy to see why they may be misconstrued as “cilia” (Figure 9A).

HR-SEM illuminates TENT development in an *in vitro* BBB model in Figure 9A. These cilia-like structures (early developing TENTs) are merely in a primordial state and, when fully developed, form TENTs [31].

Figure 9B emphatically illustrates a projecting “cilial” structure on the membrane surface of an EC [71]. Thus, we lean towards the postulate that cilia are in fact primordial versions of much more progressive TENTs.

## 3. Conclusions

The TENT and its primordial counterparts appear to be cellular protrusions that extend from the BEC membrane surface chemical stimulations. The cytoplasmic projections, both earlier (postulated cilia) and currently (primordial TENTs), appear to be identical with reference to the cytoarchitecture dynamics of the cell–cell extension features, which are governed by a cytoskeleton backbone (i.e., f-actin and α-tubulin). Furthermore, these cytoskeleton proteins act in concert to promote nanostructural TENT formation. HR-SEM studies have endorsed the observations of the transient formation of primordial TENTs, which develop into mature TENT nanostructures. Furthermore, HR-SEM scrutiny of thousands of BECs has yet to produce one observation of an authentic cilium or groups of cilia. To date, characteristic TENT features are unique to BECs. TENTs are essential for intercellular communication, facilitating BEC alignment and intercellular communication during endothelial barrier-genesis. The presence of TENTs is suggested to ensure the stability of the brain’s vascular barrier and thus it is naturally instinctive to speculate that the previously postulated cilia are in fact primordial TENTs due to their intimate molecular association and their dimension similarities. Despite BECs employing tubules as a mechanism of transcytosis, no experimental evidence has emphatically described the step-by-step documentation of BEC primary “cilia” development; however, the progression of the BEC TENT into matured tethers during BEC monolayer development has been emphatically demonstrated. TENTs are infinitesimal in nature and form transiently, and thus their morphogenesis has only been discovered recently, at HR. This review illuminates these apico-lateral structures as pivotal in occluding BEC spaces, by the formation of highly restrictive, polarized endothelial sheets during BEC monolayer formation. TENTs are suggested to be critical for angiogenesis and subsequent barrier-genesis and, thus, represent promising therapeutic targets in the treatment of cerebrovascular disorders. It is, therefore, our considered perspective that the “cilia” postulated by the earlier studies suffered from not having access to high-definition microscopy, and thus, under relatively low resolution and low magnification, these cellular projections were simply misconstrued TENT or TUNTs.

## Data Availability

The data are archived according to UWC policies. All relevant data presented in this study are available upon request from the corresponding author.

## Abbreviations

Selected Abbreviations:	
AC3	Adenylate cyclase type III
BBB	Blood–brain barrier
BEC	Brain endothelial cell
CNS	Central nervous system
EC	Endothelial cell
GFP	Green fluorescent protein
HR-SEM	High-resolution scanning electron microscopy
HR-TEM	High-resolution transmission electron microscopy
NT	Nanotubule
NV	Nanovesicle
TENT	Tethering nanotubule
TJ	Tight junction
TUNT	Tunneling nanotubule
